# Aspiration-assisted bioprinting of the osteochondral interface

**DOI:** 10.1038/s41598-020-69960-6

**Published:** 2020-08-04

**Authors:** Bugra Ayan, Yang Wu, Vengadeshprabhu Karuppagounder, Fadia Kamal, Ibrahim T. Ozbolat

**Affiliations:** 1 Engineering Science and Mechanics Department, Penn State University, University Park, PA 16802 USA; 2 The Huck Institutes of the Life Sciences, Penn State University, University Park, PA 16802 USA; 3 School of Mechanical Engineering and Automation, Harbin Institute of Technology, Shenzhen, 518055 China; 4 Center for Orthopedic Research and Translational Sciences, Department of Orthopedics and Re-Habilitation, Penn State University, Hershey, PA 17033 USA; 5 Biomedical Engineering Department, Penn State University, University Park, PA 16802 USA; 6 Materials Research Institute, Penn State University, University Park, PA 16802 USA; 7 Department of Neurosurgery, Penn State University, Hershey, PA 17033 USA

**Keywords:** Tissues, Biomaterials - cells

## Abstract

Osteochondral defects contain damage to both the articular cartilage and underlying subchon- dral bone, which remains a significant challenge in orthopedic surgery. Layered structure of bone, cartilage and the bone-cartilage interface must be taken into account in the case of biofabrication of the osteochondral (OC) interface. In this study, a dual layered OC interface was bioprinted using a newly developed aspiration-assisted bioprinting (AAB) technique, which has been the first time that scaffold-free bioprinting was applied to OC interface engineering. Tissue spheroids, made of human adipose-derived stem cells (ADSCs), were differentiated in three dimensions (3D) into chondrogenic and osteogenic spheroids, which were confirmed by immunostaining and histology qualitatively, and biochemistry assays and gene expression, quantitatively. Remarkably, the OC interface was bioprinted by accurate positioning of a layer of osteogenic spheroids onto a sacrificial alginate support followed by another layer of chondrogenic spheroids overlaid by the same support. Spheroids in individual zones fused and the maintenance of phenotypes in both zones confirmed the successful biofabrication of the histomorphologically-relevant OC interface. The biofabrication of OC tissue model without the use of polymeric scaffolds unveils great potential not only in regenerative medicine but also in drug testing and disease modeling for osteoarthritis.

## Introduction

Osteoarthritis (OA) occurs in a large amount of individuals over the age of 60, which is characterized by joint pain, swelling and stiffness^1,2^. Osteochondral (OC) defects occur most frequently in the knee, affecting the articular cartilage and subchondral bone. Painkillers and anti-inflammatory drugs are popular in treatments of OA for symptomatic relief^3^. Numerous efforts have been put to investigate new strategies to prevent or cure OA. Previous studies have identified enzymes (e.g., matrix metalloproteinases), which broke down the extracellular matrix (ECM) in the articular cartilage during OA^4^. Growth factors, such as bone morphogenetic protein 7, have been considered to enhance ECM synthesis and preserve cartilage in OA^5^. In addition, several drugs and proteins for the treatment of subchondral bone and cartilage have drawn interest including strontium ranelate, calcitonin, bisphosphonates, cathepsin K, and transforming growth factor-beta (TGFβ)^6-10^. Several models have been reported for OA drug testing in normal and pathological conditions^3^. Although there have been a plethora of advancements in drug screening, a high attrition rate still leads to tremendous time and cost suffered in pharmaceutical research^11^.

Given the high failure rate in drug discovery, development of more precise and reproducible preclinical models using advanced technologies is crucial, which closely recapitulate the native micro-environmental factors. Compared to conventional two-dimensional (2D) culture, three-dimensional culture (3D) is more favorable in imitating cell bioactivities, tissue microstructure, and cell-matrix interactions^12^. 3D bioprinting is an emerging technology that builds targeted tissues which resemble histomorphologic, micro-architectural and biomechanical properties of native ones^13-16^. Recently, several bioinks have been developed such as hyaluronic acid (HA) and chondroitin sulphate^17^. Moreover, gelatin-methacrylamide (GelMA)^18,19^, alginate^20^, and nanocellulose-alginate^21^ are some examples of biomaterials used in scaffold-based bioprinting of the OC interface. Even though significant progresses have been made, the heterogeneity of OC interface in terms of structural and biomechanical properties is difficult to recapitulate using current approaches^22^. In scaffold-based approaches, cell-cell interactions and signaling are limited as cells are confined in the scaffold matrix, which is essential for cell differentiation and mechanotransducive signaling between cells in order to regenerate the OC interface with heterogenic structural, biological and mechanical characteristics^23^. Tissue strands, honeycombs, cell aggregates or spheroids are some examples of bioink used for scaffold-free biofabrication of tissues^24^.

The OC tissue, is constituted of bone, cartilage and the interface, which is not trivial to manufacture easily because of its heterogeneous and anisotropic architecture^22,25^. Aspiration-assisted bioprinting (AAB), which is recently reported by our group, is a novel method for precise positioning of viscoelastic tissue spheroids in both scaffold-free and scaffold-based manners^26^. Spheroids can be picked and lifted into air by aspiration forces, which can then be bioprinted onto a sacrificial hydrogel (alginate) at remarkable placement precision, ~ 11% with respect to the diameter of spheroids. At the end, the bioprinted construct are overlaid with alginate for culture. Taking the advantage of accurate positioning of tissue spheroids, this study is the first time to resemble the human OC interface using 3D scaffold-free bioprinting. This bottom-up approach with high precision renders a compact and stratified tissue arrangement, which is more biomimetic in terms of cell density and ECM organization, as compared to previously reported methods^27^. AAB enabled picking of spheroids from the cell media with aspiration forces and bioprint them onto/into hydrogel substrates without any damage. In this study, patient-derived adipose-derived stem cells (ADSCs) were used to fabricate spheroids, which were then differentiated towards osteogenic and chondrogenic lineages in three weeks. Osteogenic and chondrogenic spheroids were then 3D bioprinted in multiple zones and then self-assembled into a single patch of tissue, which recapitulated the OC interface with histologically-relevant morphologies. The bioprinted OC interface has the potential to be used not only in tissue engineering and regenerative medicine but also in OA disease modeling for drug testing and the development of new therapeutic strategies.

## Results

### Formation of spheroids

**Figure 1:**
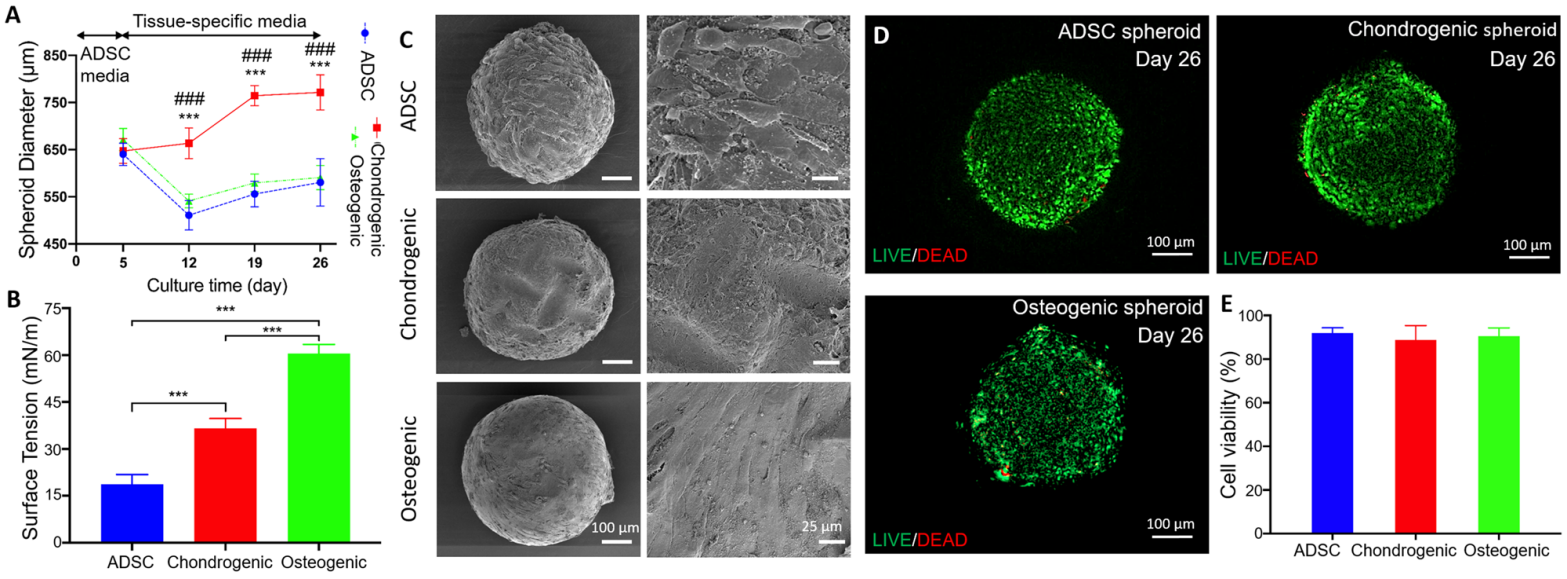
Structural, mechanical, and cell viability analysis of spheroids: (**A**) Diameter change over 26 days (*n* = 8,***p < 0.001 shows significance between ADSC and chondrogenic spheroids, and ###*p* < 0.001 shows significance between osteogenic and chondrogenic spheroids), (**B**) surface tension at Day 26 (*n* = 5, ***p < 0.001), and (**C**) SEM images of ADSC, chondrogenic and osteogenic spheroids. (**D**) LIVE/DEAD images (**E**) and cell viability in ADSC, chondrogenic, and osteogenic spheroids at Day 26.

In this study, ADSC spheroids were obtained with a density of 2 × 10^4^ cells/spheroid using 96 U-shape well-plates. After five days of culture, formation of compact ADSC spheroids were observed. Afterwards, spheroids were cultured with osteogenic or chondrogenic induction media for 21 days before bioprinting the OC interface. The average diameter of ADSC spheroids were ~ 650 µm at Day 5 (Fig. 1A). One week after differentiation, the average diameter of ADSC, chondrogenic and osteogenic spheroids were measured to be ~ 510, 663, and 541 µm, respectively. In another words, the average diameter of ADSC and osteogenic spheroids decreased by ~ 20 and 19% at Day 12, respectively. However, no significant changes were observed for the average diameter of chondrogenic spheroids. After the second week of differentiation, the average diameter of ADSC, chondrogenic, and osteogenic spheroids were measured to be ~ 555, 764, and 580 µm at Day 19, respectively, in which the average diameter of chondrogenic spheroids displayed an increase of ~ 15%. Overall, the average diameter of ADSC and osteogenic spheroids decreased by ~ 9 and 13% at Day 26, respectively, while, the average diameter of chondrogenic spheroids increased by ~ 19% during the prolonged culture time, with significant differences (***p < 0.001 shows significance between ADSC and chondrogenic spheroids, and ###*p*<0.001 shows significance between osteogenic and chondrogenic). The surface tension of ADSC, chondrogenic and osteogenic spheroids, representing the mechanical properties of viscoelastic spheroids, were measured to be ~ 19, 37, and 60 mN/m, respectively (Fig. 1B). After 26 days of culture, all groups exhibited compact morphologies (Fig. 1C). After the differentiation of each tissue type, cell viability was investigated at Day 26, which could be considered long term. LIVE/DEAD staining demonstrated a high level of cell viability (Fig. 1D), which was further confirmed by the quantitative measurements, showing ~ 92, 94 and 96% of live cells in ADSC, chondrogenic and osteogenic spheroids, respectively, without significant difference (*p* > 0.05, Fig. 1E).

### Qualitative and quantitative analysis of chondrogenic and osteogenic spheroids

**Figure 2:**
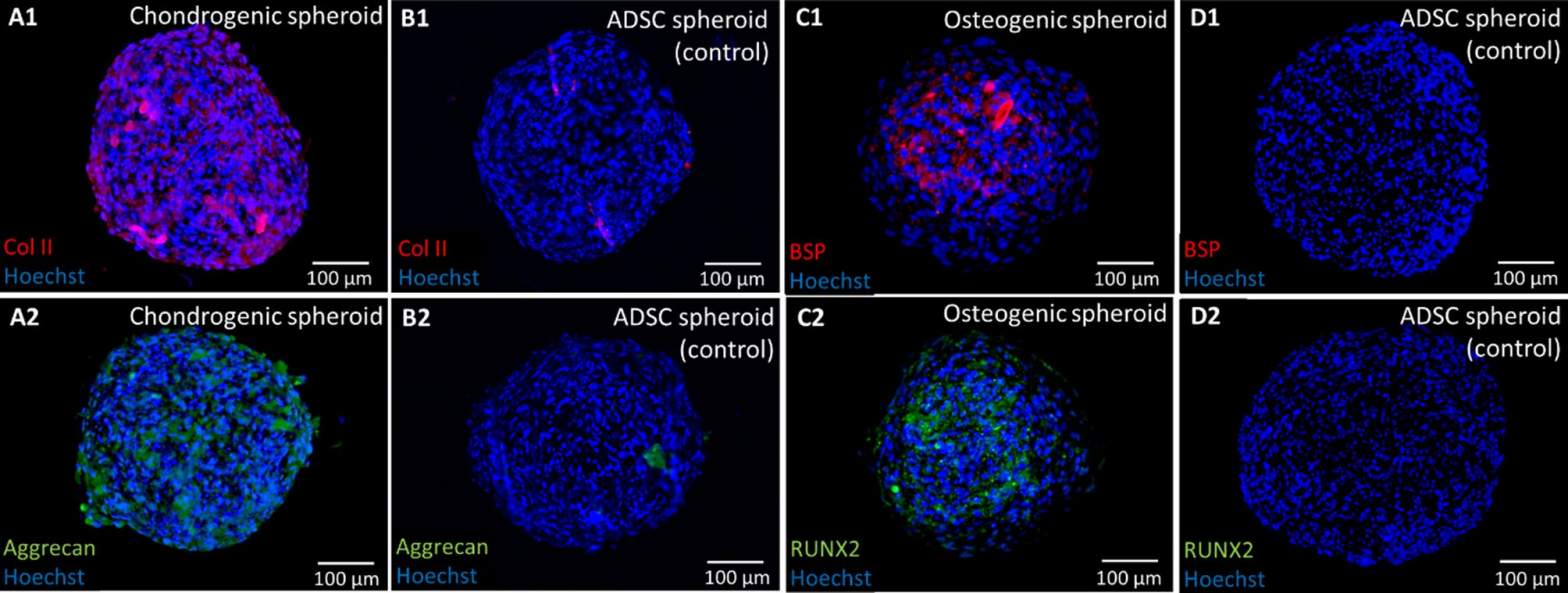
Expression of cartilage-specific markers including (**A**) COL-II and (**B**) Aggrecan for chondrogenic and ADSC spheroids, and bone-specific markers including (**C**) BSP and (**D**) RUNX2 for osteogenic and ADSC spheroids.

In order to fabricate the OC interface, ADSC spheroids were differentiated into chondrogenic and osteogenic lineages. Immunofluorescence staining was conducted to evaluate the expression of cartilage-specific markers, in order to verify the chondrogenesis of ADSC spheroids (Fig. 2A1–B2). Images show substantial expression of COL-II and Aggrecan distributed throughout the chondrogenic spheroids as compared to the ADSC control. The results demonstrated that ADSCs were able to chondrogenically differentiate in the form of spheroids and exhibit chondrogenic phenotype. Similarly, osteogenesis was evidenced by the superior expression of bone-specific markers, including the early-stage marker RUNX2 and late-stage marker BSP, in osteogenic spheroids as compared to ADSC spheroids (Fig. 2C1–D2).

**Figure 3:**
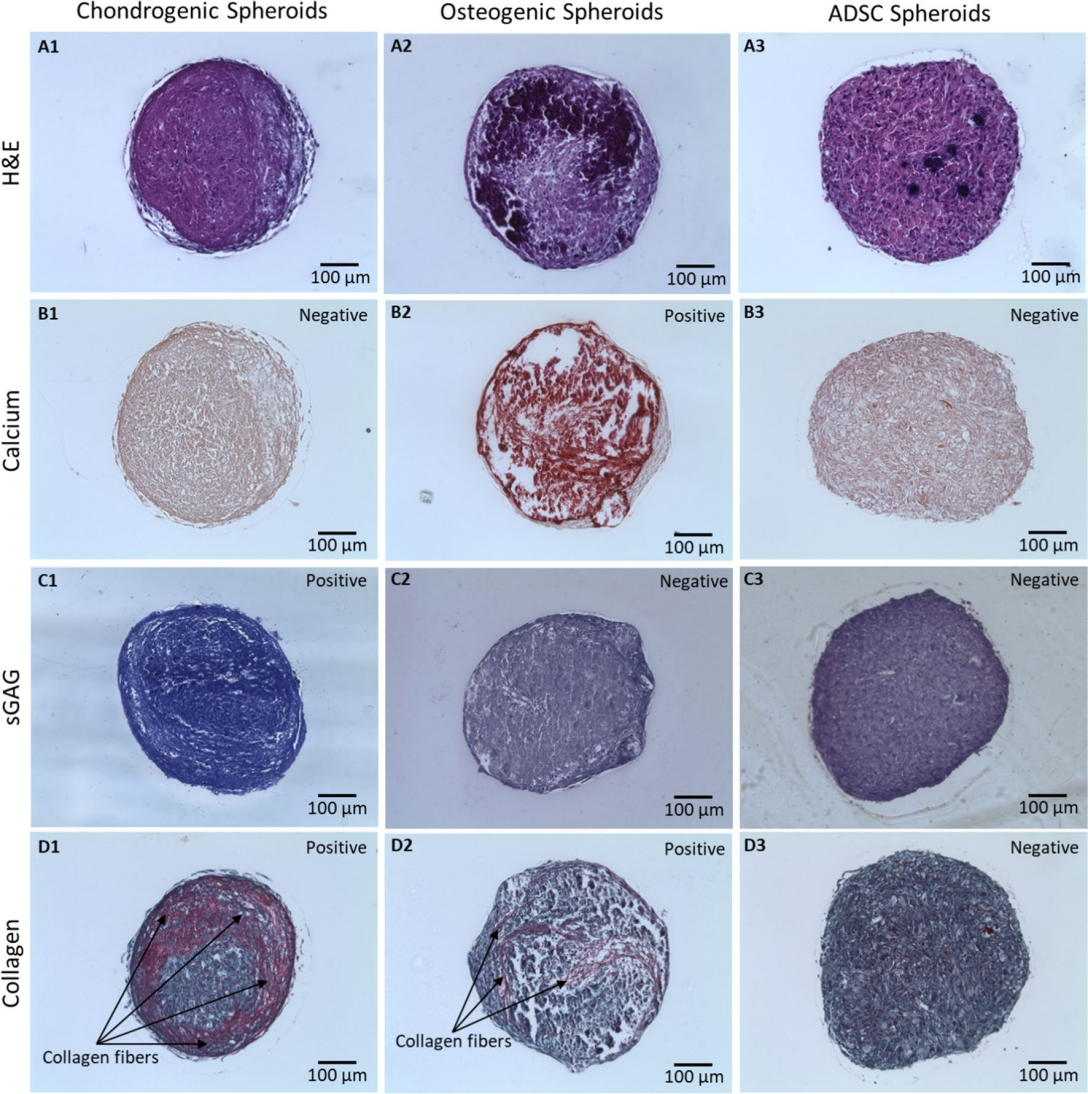
Histological staining of ADSC, osteogenic, and chondrogenic spheroids for (**A**) H&E, (**B**) Alizarin Red, (**C**) Toluidine Blue, and (**D**) Picrosirius Red/Fast Green.

H&E images show that cells in all groups were densely packed without any visible necrotic core (Fig. 3A). Particularly, for osteogenic spheroids, higher cell density and ECM deposition were observed around the surface of spheroids. In contrast, chondrogenic spheroids demonstrated more homogeneous cell distribution of cells compared to their osteogenic counterparts. Osteogenic spheroids exhibited a strongly positive signal for Alizarin Red indicating the abundance of calcification, as compared to ADSC and chondrogenic spheroids (Fig. 3B). Toluidine Blue staining of chondrogenic spheroids showed violet color demonstrating higher level of sulfated glycosaminoglycan (sGAG) deposition while ADSC and osteogenic were negative (Fig. 3C). In addition, a limited amount of collagen was observed in ADSC spheroids (Fig. 3D). In contrast, a moderate level of collagen fibers was identified in osteogenic spheroids. Chondrogenic spheroids exhibited dense and thick collagen fibers, especially around the edge of the spheroids.

**Figure 4:**
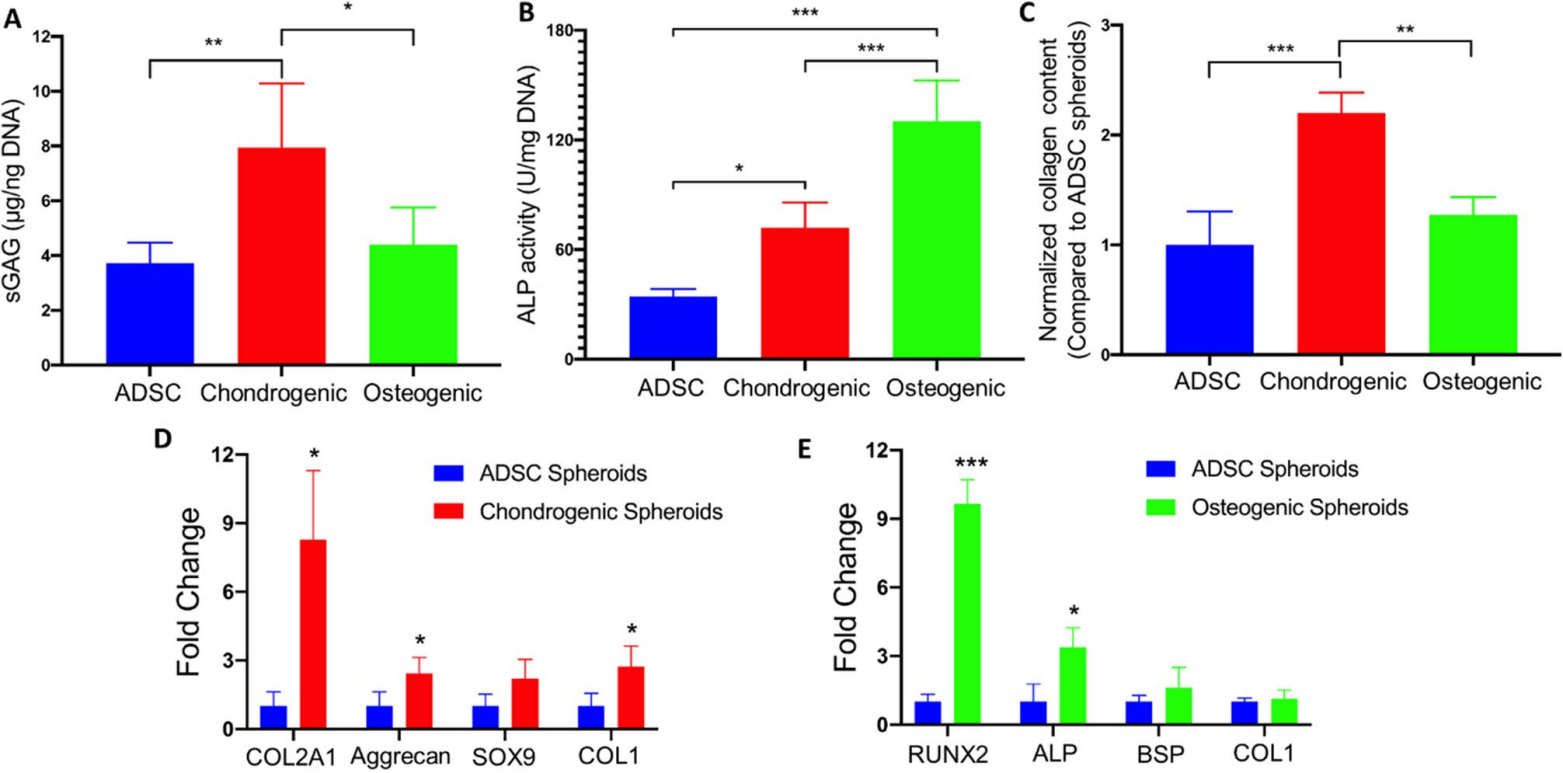
Quantification of protein and gene expression in different types of spheroids. (**A**) sGAG content measurement normalized to the DNA amount (*n* = *3*). (**B**) ALP activity normalized to DNA amount (*n* = *3*). (**C**) Collagen deposition. RT-PCR analysis demonstrating the expression of (**D**) cartilage- and (**E**) bone-specific genes (*n* = 3 for all analysis*,* *p < 0.05, **p < 0.01, and ***p < 0.001).

The chondrogenic and osteogenic induction of ADSCs in the 3D spheroid culture was further confirmed quantitatively. Chondrogenic spheroids yielded significantly greater dsDNA normalized sGAG content as compared to ADSC and osteogenic spheroids (with ~ 2.1 and 1.8-fold increase, respectively, *p* < 0.05, Fig. 4A). In regards to osteogenesis, the biochemical assessment showed that ALP activity of osteogenic spheroids was significantly superior with respect to both ADSC and chondrogenic spheroids with a ~ 3.8 and 1.8-fold increase, respectively (*p* < 0.05, Fig. 4B). The collagen expression analysis revealed that chondrogenic and osteogenic spheroids possessed higher collagen matrix deposition than ADSC spheroids. Chondrogenic spheroids demonstrated a ~ 2.2-fold increase in collagen expression compared to ADSC spheroids, while osteogenic spheroids showed only a ~ 1.3-fold increase, which was consistent with the Picrosirius Red staining results.

In terms of cartilage-specific gene expression, chondrogenic spheroids had a significantly greater expression of COL2A1, Aggrecan and COL1 (~ 8.3, 2.4 and 2.7-fold increase, respectively, *p* < 0.05) than the ADSC group (Fig. 4D). In addition, the gene expression of SOX9 in chondrogenic spheroids revealed a ~ 2.2-fold increase against the ADSC group. In terms of bone-specific gene expression, osteogenic spheroids exhibited significantly superior gene expression of RUNX2 and ALP (~ 9.6 and 3.4-fold increase, respectively, *p* < 0.05, Fig. 4E). Meanwhile, greater expression of BSP and COL1 as compared to the ADSC group was observed. These results demonstrate that chondrogenic and osteogenic induction happened in ADSC spheroids after a three-week induction of differentiation media in the 3D spheroid culture.

### 3D bioprinting of the OC interface

**Figure 5:**
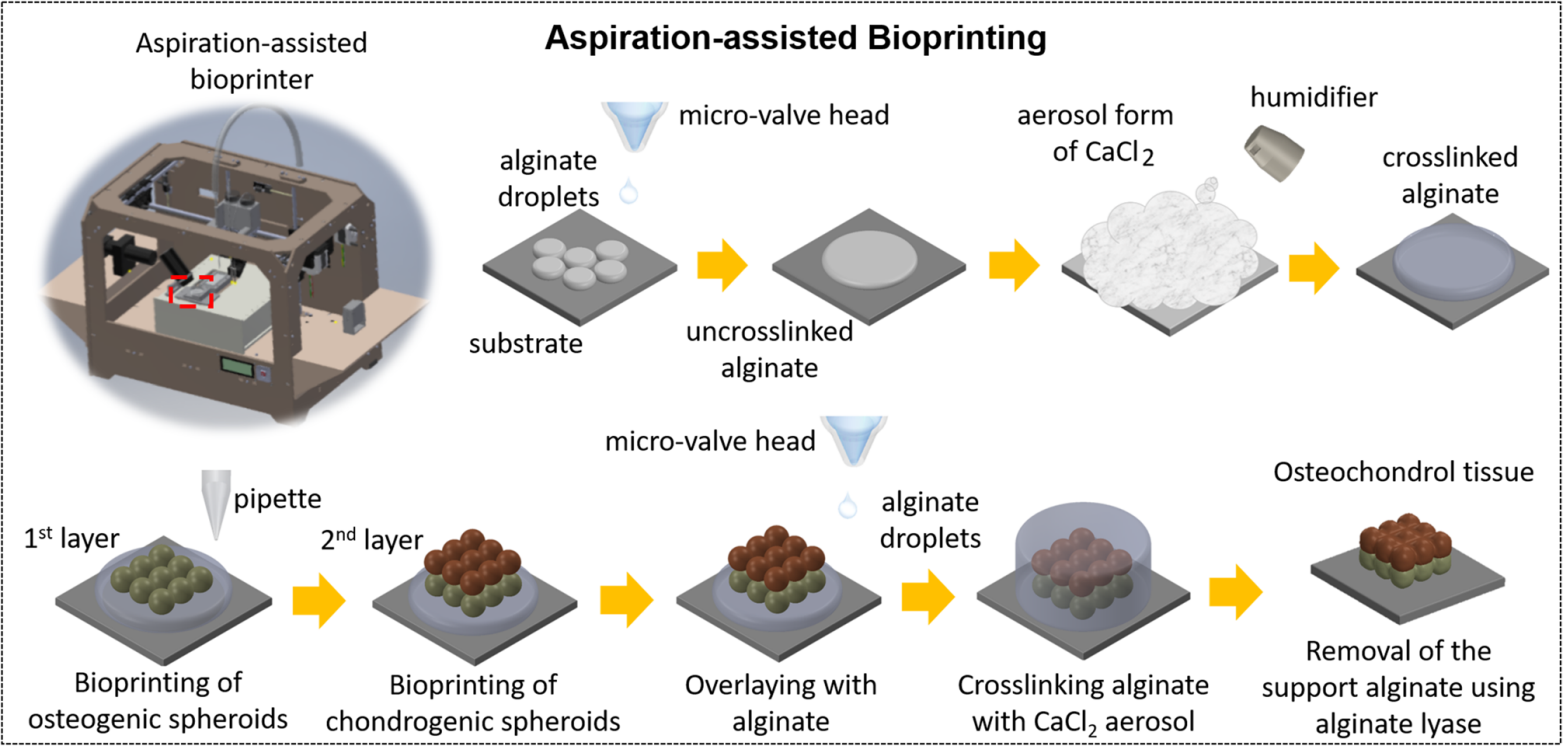
A schematic illustration showing the aspiration-assisted bioprinting (AAB) of the OC interface with chondrogenic and osteogenic zones.

In this study, a newly developed AAB process^26^ was utilized to fabricate the OC interface. AAB facilitated precise positioning of viscoelastic tissue spheroids in 3D, and when combined with micro-valve bioprinting, it enabled the self-assembly of these spheroids in a sacrificial alginate support. Being the first step of process (Fig. 5), a spheroid was picked, lifted and dragged rapidly outside the culture media using aspiration. The back pressure was cut off when the spheroid was transferred onto the bioprinting stage. Spheroids were partially submerged into the partially-crosslinked alginate support since pushing them further could lead to pipette tip penetrating into the spheroid. When the nozzle moved up, the spheroid was deposited due to adherence between the spheroid and alginate. The procedure was repeated as many times as needed in order to build the OC interface. At the last step, bioprinted constructs were overlaid with alginate using micro-valve bioprinting and calcium chloride (CaCl_2_) vapor was then applied to crosslink alginate. After bioprinting of spheroids, the construct was maintained in the alginate support for a week to facilitate complete fusion of spheroids and alginate was then de-crosslinked leaving the assembled OC interface behind. In order to successfully bioprint of the OC interface, we utilized aspiration pressure of 95 and 74 mmHg for osteogenic and chondrogenic spheroids, respectively, as these pressure levels were sufficient to transfer spheroids and did not induce major stress on them that could result in their complete aspiration, breakage, or substantial cell death.

**Figure 6:**
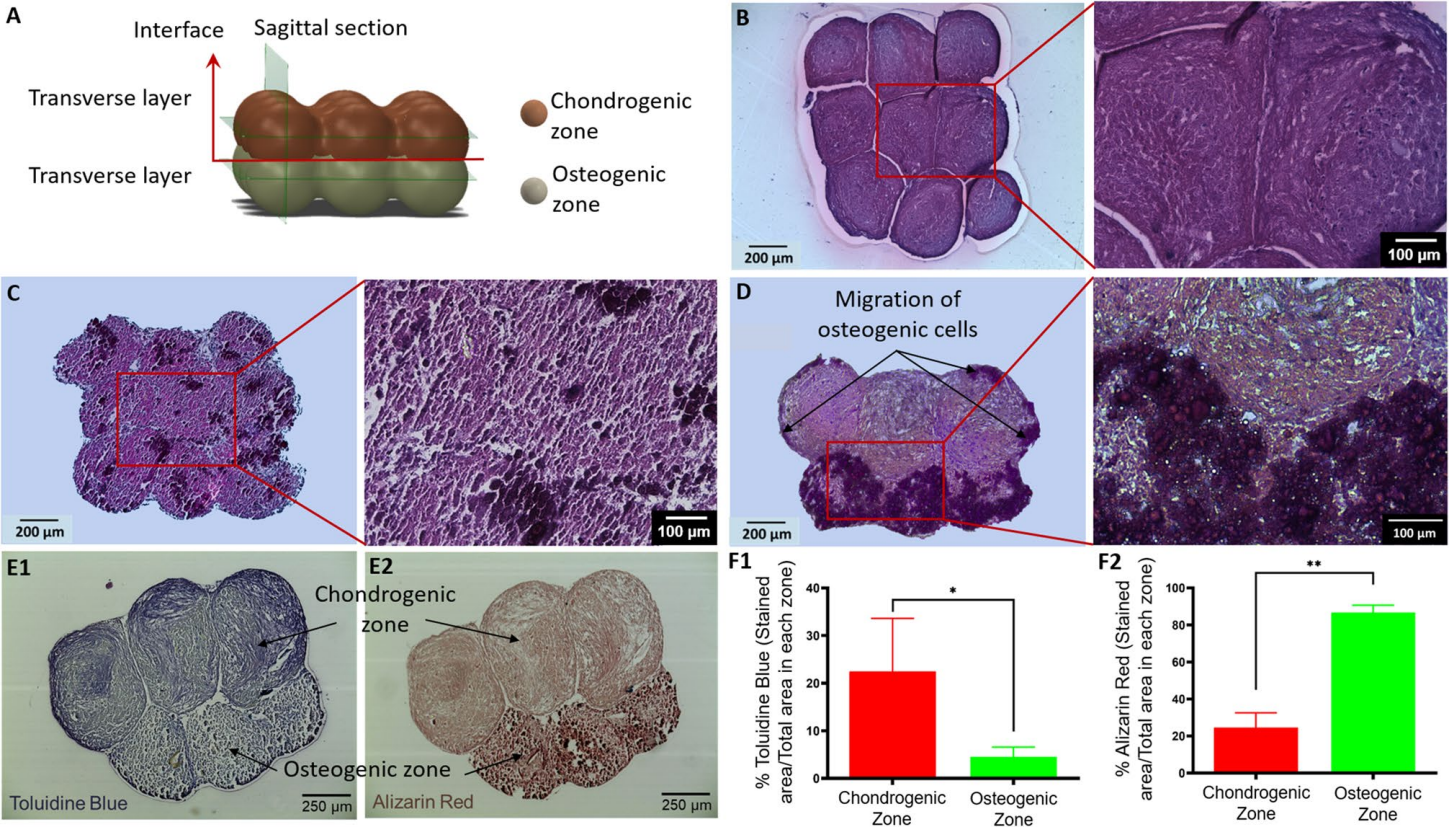
(**A**) A schematic diagram showing positions of histological sectioning in the construct. (**B-D**) H&E staining of the chondrogenic, and osteogenic zones, and the interface. Histomorphological characteristics of the bioprinted OC interface indicated by (**E1**) Toluidine Blue and (**E2**) Alizarin Red staining. Quantification of stained areas for Toluidine Blue (**F1**) and Alizarin Red (**F2**) in chondrogenic or osteogenic zones (*n* = 4*,* *p < 0.05 and **p < 0.01).

The OC interface was bioprinted and then sectioned according to the model given in Fig. 6A in order to visualize the chondrogenic and osteogenic zones and the interface. The cross-sections of chondrogenic and osteogenic zones in images of hematoxylin and eosin (H&E) straining exhibited compact tissue with fused spheroids in a 3 × 3 arrangement (Fig. 6B–D). Similar to the uniform morphology of individual spheroids, the chondrogenic zone also showed more uniform cellular and ECM distribution as compared to the osteogenic layer. Most importantly, chondrogenic and osteogenic zones fused completely with the absence of gaps in between (Fig. 6D). In addition, both zones were apparently distinguishable with osteogenic zone showing darker purple color and chondrogenic zone displaying more even matrix, demonstrating the maintenance of their respective phenotypes after fusion.

In order to confirm the histomorphological characteristic of the OC interface, Toluidine Blue and Alizarin Red staining were performed. As expected, the chondrogenic zone was dyed with violet color showing expression of sGAG, when it compared to the pale staining on the osteogenic zone (Fig. 6E1). The osteogenic zone exhibited ample deposition of calcium component, which was indicated by the dark orange color in Alizarin Red staining (Fig. 6E2). Further quantification based on area measurement revealed that Toluidine Blue strained area in the chondrogenic zone (~ 22%) was significantly greater than that in the osteogenic zone (~ 5%, *p* < 0.05, Fig. 6F1). On the contrary, Alizarin Red stained area in the osteogenic zone (~ 86%) outnumbered that in the chondrogenic zone (~ 24%, *p* < 0.05, Fig. 6F2).

## Discussion

In this study, a double-layer OC interface was 3D bioprinted using the AAB technique, which mimics the structure of subchondral bone and articular cartilage in native tissue^28^. The OC interface is of importance in transfer of forces and retaining the integration of bone and cartilage ^29^. The OC tissue exhibits a thickness of ~ 3 mm in adults^30^, and the chondrogenic and osteogenic zones in 3D bioprinted tissues had a thickness of ~ 1 mm, which was in the same order of magnitude as the native OC interface.

Chondrocytes and osteoblasts are popular cell sources for studies on osteochondral repair^22^. However, the limited amount of chondrocytes and osteoblasts in the body, comprising of 2% of the cartilage volume and 4-6% of total resident bone cells, respectively, restrained their translational applications^31-33^. Particularly, chondrocytes might be destroyed during extraction process since digestion by collagenase is reqiuired^34^. Most importantly, although adult chondrocytes are capable of cell division in tissue culture, they exhibit telomere shortening and dysfunction after multiple passages, which is related to replicative senescence and senescent secretory phenotype^35^. Hence, an alternative and readily available cells is desired for the OC repair. In this study, ADSCs were used as a cell source, which are convenient to harvest as compared to mature cells and other adult stem cells^36^.

In terms of surface tension that represents the mechanical properties of viscoelastic spheroids^26^, the highest value was observed for osteogenic spheroids, which could be attributed to the mineralized surface compactness of spheroids. Chondrogenic and osteogenic spheroids exhibited more compact organization as compared to ADSC spheroids, which could be because of the abundant ECM production on the differentiated spheroids^37^. It has been well acknowledged that 3D culture is more sufficiently mimic the in vivo tissue formation with regard to the level of differentiation as compared to 2D culture^38^. Especially for chondrocytes, 3D culture prevented them from dedifferentiation and preserved their chondrogenic phenotype^39^. In addition, the formation of osteogenic spheroids using differentiated ADSCs or ADSCs undergoing osteogenic differentiation was not quite possible in our initial experiments as cells did not tend to assemble into spherical aggregates (data not shown here). Therefore, we preferred to differentiate the cells in the form of spheroids. As hypoxia usually occurs in cell aggregates with diameter over 500 μm^40^, we formed ADSCs spheroids with a diameter of ~ 500 μm. The successful differentiation of ADSCs in the form of 3D spheroids were proven by immunostaining, in which COL II and Aggrecan for chondrogenesis and BSP and RUNX2 for osteogenesis were present throughout the spheroids. Similar results in histological straining were also observed, with calcium component presented in osteogenic spheroids, sGAG expressed in chondrogenic spheroids, and collagen fibers observed in both. The sGAG/DNA result in this study was comparable to previously published cell aggregate-based studies^41,42^, and much higher than the results in scaffold-based studies^42,43^. Such a high sGAG/DNA content can be attributed to the low amount of DNA as well as higher cell density in spheroids compared to that in cell-laden hydrogels resulting in up-regulated ECM deposition owing to close cell-cell interactions. More uniform cell distribution was observed in chondrogenic spheroids compared to other two groups due to the anaerobic nature of chondrocytes as consistent with our previous findings^40^. The presence of live cells and abundant amount of ECM in the core of spheroids indicated the absence of hypoxic core and the diffusion of induction media in the spheroids. In addition, the quantitative results, including biochemistry and RT-qPCR, further confirmed that differentiated phenotypes were maintained.

In the context of OC tissue engineering, scaffolds, which displayed either discrete or continuous gradients, have been reported^22,31,44^. Besides naturally-derived materials including collagen type I and II, hydroxyapatite, GAG, HA, gelatin and chondroitin sulfate, other synthetic materials, such as poly(lactic-co-glycolic acid), polycaprolactone (PCL) and poly(ester-urethane), have been widely used to resemble the bone and cartilage compartments^45-54^. With the advances in bioprinting, investigations of bioprinted OC constructs have been reported recently. Levato et al^19^. loaded mesenchymal stem cells (MSCs) within microcarriers of polylactic acid (PLA), and subsequently encapsulated cell-laden microcarriers in GelMA-gellan gum (GG). The GelMA-GG was used to bioprint cartilage zone, while microcarrier-encapsulated GelMA-GG represented the bone zone. Being a supporting frame, PCL was used for extrusion of hydrogel-laden human MSCs in a study reported by Shim and collegues^55^. The subchondral bone was bioprinted using MSCs-laden atelocollagen and recombinant human bone morphogenic protein (rhBMP)-2, while the cartilage was bioprinted with MSCs, hyaluronic acid and transforming growth factor beta (TGF-β). Different from the above-mentioned scaffold-based strategies, the OC interface presented in this work was successfully reconstitued using a scaffold-free strategy, which was comprised of single layer of chondrogenic spheroids and another layer of osteogenic spheroids in a 3 × 3 matrix arrangement. Cell density in scaffold-based methods is usually in the order of 10^6^ cells/mL^56-58^, while it can reach 10^7^–10^8^ cells/mL equivalently in scaffold-free tissues due to the absence of scaffolding hydrogels^40^. The spheroids were bioprinted in a pick-and-place fashion using the AAB bioprinter, to accurately position them ensuring their successful fusion to form a well-defined interface arrangement. Since the spheroids were merely comprised of cellular components, considerations in scaffold-based methods, such as the choice of biomaterials, material degradation and cell-matrix interactions, were no longer concerns. Interestingly, osteogenic cells were observed at the edge of the chondrogenic zone in some of the bioprinted OC interface samples (Fig. 5E). This observation might be attributed to the migration of cells from the bone zone to the cartilage zone due to the use of chondrogenic and osteogenic media with 1:1 ratio, resulting in the calcification of chondrogenic zone. Such interaction could probably be a model for investigation for calcification of articular cartilage, which is commonly observed during aging or osteoarthritis^59^. In addition, the determination of bioactivity for each zone is quite challenging in the bioprinted OC interface. Therefore, we performed bioactivity assessment of single spheroids before bioprinting as they could be representative for the induvial zones in the bioprinted OC interface.

Since healthy human articular chondrocytes are difficult to obtain because of ethical issues, in vitro and in vivo models have been investigated to explain the molecular mechanisms in joint pathophysiology^3^. In addition, interaction between bone and cartilage cells plays a key role in driving OA pathological processes, which was evidenced by the co-culture of osteoblasts and chondrocytes isolated from the OA subchondral bone and cartilage^60^. The bioprinted OC interface in this study contained osteogenic and chondrogenic zones with high cell density enabling close cell-cell interactions and histomorphologically-relevant of 3D interface arrangement, which offers great potential of the presented OC interface for use in disease modeling and drug testing.

Herein, it has been the first time that an OC interface was bioprinted using a scaffold-free methodology. After chondrogenesis and osteogenesis in a 3D spheroid culture, differentiated ADSC spheroids exhibited cartilage- and bone-specific gene and protein expressions, which were then precisely bioprinted to generate a double-layer arrangement with the top and bottom zone imitating the cartilage and bone, respectively. The bioprinted OC interface exhibited a tightly-integrated organization between chondrogenic and osteogenic zones, with the preservation of their histomorphological characteristic in terms of tissue-specific protein deposition. This study has opened a new avenue for the biofabrication of the OC interface model without the use of polymeric scaffolds, which has great potential to be utilized not only in regenerative medicine and tissue engineering but also in drug testing and disease modeling.

## Methods

### Spheroid preparation and differentiation

Human ADSCs was obtained according to the protocol reported as our previous work^40^. Surgically discarded adipose tissues were obtained from patients who underwent an elective adipose tissue removal process (e.g., panniculectomy) at the Pennsylvania State University (Hershey, PA). The human study was approved by the Pennsylvania State University Institutional Review Board (protocol #4972). Patients’ informed consent was obtained for study participation. All methods were performed in accordance with the relevant guidelines and regulations. ADSCs were isolated according to the protocol described in our previous work with verification of flow cytometry against CD73 and CD90^40^. The sorted ADSCs were cultured in DMEM/F12 supplement with 20% FBS, 100 U/mL penicillin and 100 µg/mL streptomycin at 37 °C with 5% CO2. Cell medium was changed every three days. Expanded ADSCs were then used for the preparation of spheroids. ADSCs were trypsinized and centrifuged to cell pellets. Cells were then suspended in the media to obtain a density of 1 × 10^6^ cells/mL. Afterwards, 200 µL of cell suspension per well was transferred to a 96-well plate (Greiner Bio One, NC) in order to obtain an average density of ~ 2 × 10^4^ cells per well. After 5-day culture with ADSC growth media, the growth media was replaced with Human Chondrocyte Differentiation Media and Human Osteoblast Differentiation Medium (Cell Applications, CA) for chondrogenic and osteogenic differentiation of spheroids for another 21 days, respectively. As a control group, ADSC spheroids were also cultured for another 21 days using the ADSC growth media.

### Determination of the physical properties of spheroids

Spheroids were cultured in differentiation media from Day 5 and the diameters of spheroids were measured by an EVOS FL cell imagining system (Life Technologies) until Day 26 according to our previous work^26^. Surface tension of spheroids was also measured according to the protocol described in our recently published work^61^. Briefly, after collecting the spheroids from well plates, they were washed with phosphate buffer saline (PBS) for three times. Customized straight micropipettes, fabricated from glass pipettes (VWR, PA) using a P1000 Flaming/Brown micropipette puller (Sutter Instrument, CA), were used for surface tension measurements. Aspirated spheroids were monitored via a STC-MC33USB monochromatic camera (Sentech, Japan). The surface tension for ADSC, chondrogenic and osteogenic spheroids were measured at Day 26.

### Scanning electron microscope (SEM) imaging

Field Emission SEM (FEI Navo NanoSEM 630, Nanolab Technologies, CA) was used to probe the surface topography of fabricated ADSC, chondrogenic, and osteogenic spheroids. Spheroids were fixed in 4% paraformaldehyde (Santa Cruz Biotechnology, TX) overnight, carefully washed with PBS, and dehydrated using graded ethanol solutions (25%, 50%, 75%, 95% and 100%, sequentially) following the protocol reported in our previous work^40^. To ensure the complete removal of water, samples were further dried in a critical point dryer (CPD300, Leica EM, Germany). Upon complete dehydration, they were sputter coated with iridium (Leica, Germany) and imaged at an accelerating voltage of 3-5 keV.

### Cell viability analysis

ADSC, chondrogenic, and osteogenic spheroids were collected from 96-well plates, and cell viability was assessed using LIVE/DEAD viability assay kit (Life Technologies, NY)^26^. First, spheroids were washed for three times using PBS and then incubated in a fluorescent reagent consisting of 1 μM calcein AM and 1.6 μM ethidium homodimer-1 in PBS for 30 min. Live cells were able to take up and retain the calcein dye resulting in bright green fluorescence of their cytoplasm. The ethidium homodimer could only enter dead cells, where it binds to nucleic acids producing a bright red fluorescence. Z-stack images were taken on the EVOS FL cell imagining system. ImageJ software (National Institute of Health, USA) was used for quantitative analysis for red- and green-fluorescent cells.

### Immunofluorescence study

All primary monoclonal antibodies were purchased from Abcam (MA) and fluorescence-conjugated secondary antibodies were purchased from Life Technologies (CA). Immunofluorescent staining was performed according to the protocol reported in our previous work^40^. Sections of ADSC and chondrogenic spheroids were treated using Triton-X 100 (0.1 % in PBS) for 10 min and blocked with normal goat serum (NGS, 10 % in PBS) for 1 h. Samples were then incubated with monoclonal rabbit anti-human collagen type II (COL-II, 1:200), mouse anti-human aggrecan (1:50) and NGS (negative control) for 1 h, respectively. Samples were washed twice with PBS and incubated using secondary antibodies (goat anti-rabbit IgG (H+L)-Alexa Fluor 647 for COL-II, and goat anti-mouse IgG (H+L)-Alexa Fluor 488 for aggrecan, both at 1:200 dilution) for 1 h. After rinsing thrice with PBS, samples were incubated with Hoechst 33258 (1:200, 5 min) for nucleus visualization and mounted with Fluoromount-GTM (Invitrogen, MA). Using the same protocol, sections of ADSC and osteogenic spheroids were stained for bone-specific primary antibodies, including rabbit anti-human bone sialoprotein (BSP, 1:200) and runt-related transcription factor 2 (RUNX2, 1:200), followed by incubation with the secondary antibodies (goat anti-rabbit IgG (H+L)-Alexa Fluor 647 for BSP, and goat anti-mouse IgG (H+L)-Alexa Fluor 488 for RUNX2, both at 1:200 dilution). Images for each marker were taken using a Zeiss Axiozoom microscope (Carl Zeiss Microscopy, LLC, Germany).

### Histological analysis

ADSC, Chondrogenic, and osteogenic spheroids were fixed with 4% paraformaldehyde and embedded in paraffin using an automatic tissue processor (TP 1020, Leica, Germany). Next, spheroids were gradually dehydrated in alcohol, cut into 8 µm sections, and placed onto charged slides. Sections were then stained with H&E using Leica Autostainer XL (Leica, Germany). Coverslips were mounted on the slides with Xylene Substitute Mountant (Thermo Fisher Scientific, PA)^40^.

Sections were also separately stained for Alizarin Red S, Toluidine Blue O, and Picrosirius Red according to manufacturers’ protocols for detection of calcium, sulfated proteoglycan, and collagen, respectively. For Alizarin Red S staining, sections were incubated with Alizarin Red solution (EMD Millipore Corp., USA) at room temperature for 20 min. The dye was removed and dehydrated with acetone and acetone-xylene (1:1) solutions, sequentially. Samples were then cleaned with xylene and mounted with Xylene Substitute Mountant. For Toluidine Blue O staining, sections were incubated in a Toluidine Blue solution (0.1% in deionized (DI) water) (Sigma Aldrich, MO) at room temperature for 2 min. The dye was removed and then the samples were washed twice with DI water. After dehydration with 95% and 100% alcohol (sequentially) and clearing with xylene, coverslips were mounted to slides. For Picrosirius Red staining, the solution was prepared by dissolving 0.1% Direct Red 80 and 0.1% Fast Green FCF dissolved in saturated aqueous picric acid (1.2% picric acid in water, Sigma Aldrich, MO). The solution was then applied to sections and then the sections were incubated for 60 min. Samples were rinsed with DI water, dehydrated with alcohol, cleaned with xylene, and then mounted. Finally, samples were imaged using the EVOS FL cell imagining.

### Biochemical assays

For samples undergoing chondrogenic differentiation, sulfated glycosaminoglycan (sGAG) content was determined by 1,9-dimethylmethylene blue (DMMB) dye-binding assay^40^. For each sample, 10 chondrogenic spheroids were collected and rinsed with PBS. Next, spheroids were digested in 500 μL solution of 0.1 mg/mL papain extraction reagent at 65 °C for 18 h. 20 µL of digested samples were mixed with 200 µL DMMB solution and the absorbance was measured at 525 nm using a microplate reader (PowerWaveX, BioTek, VT). A series of solution of chondroitin 4 sulfate was prepared as a standard and sGAG contents of samples were calculated according to the standard curve. For samples undergoing osteogenic differentiation, alkaline phosphatase (ALP) activity assay was conducted using an assay kit (K412-500; BioVision, Inc., CA) according to the manufacturer's instructions^40^. 10 spheroids per sample were re-suspended in an assay buffer and subsequently centrifuged at 13,000*g* for 3 min at 4 ˚C to remove the insoluble material. The supernatant was mixed with p-nitrophenyl phosphate (pNPP) substrate and then incubated at 25 ˚C for 60 min. The optical density of the resultant pNPP at 405 nm was determined at 405 nm.

In order to determine the total collagen content of each spheroid type, a hydroxyproline colorimetric assay kit (BioVision, BioVisision Inc, CA) was utilized according to our previous paper^26^. Fifty spheroids per sample were collected at Day 26 and washed with DPBS. Afterwards, samples were homogenized in 100 µL of distilled water and transfer to tight vials. 100 µL of 12 N hydrochloric acid was added and incubated at 120 ˚C for 3 h. All measurements were taken using a Powerwave X-340 spectrophotometer (Biotek, Winooski, VT) at 560 nm and the results were normalized to the collagen amount expressed by the ADSC group.

A Quant-iTTM PicoGreen dsDNA Assay Kit (Molecular Probes Inc., OR) was used to determine the DNA amount, according to the manufacturer’s instructions^40^. Fluorescence intensity was determined by a SpectraMax multi-detection microplate reader (Molecular Devices, Inc., CA), using a wavelength of 480 nm (excitation) and 520 nm (emission). sGAG content and ALP activity were normalized to dsDNA content.

### Gene expression study using quantitative real-time polymerase chain reaction (RT-qPCR)

In order to evaluate cartilage- and bone-specific gene expression levels, TR-qPCT was performed according to our previous paper^40^. 30 differentiated spheroids per sample at Day 26 were homogenized in TRIzol reagent (Life Technologies, CA), followed by adding 0.2 mL chloroform per 1 mL TRIzol reagent and centrifuging the mixture at 12,000*g* for 15 min at 4 ˚C. The upper aqueous phase with RNA was transferred and the RNA was then precipitated by adding 0.5 mL isopropyl alcohol per 1 mL TRIzol reagent, followed by centrifuging at 12,000*g* for 10 min, at 4 ˚C. Subsequently, the precipitated RNA was rinsed twice by 75% ethanol, air-dried for 10 min and dissolved in 50 µL diethyl pyrocarbonate (DEPC)-treated water. RNA concentration was measured using a Nanodrop (Thermo Fisher Scientific, PA). Reverse transcription was performed using AccuPower® CycleScript RT PreMix (BIONEER, Korea) following the manufacturer's instructions. Gene expression was analyzed quantitatively with SYBR Green (Thermo Fisher Scientific, PA) using a 7500 RT-PCR system (Applied Biosystems®, Life Technologies, USA). Cartilage-specific genes tested for chondrogenic spheroids included collagen type II (COL2A1), Aggrecan, collagen type I (COL1), and the chondrogenic transcription factor SOX9. Bone-specific genes tested for osteogenic spheroids included BSP, RUNX2, COL1, and ALP. The reader is referred to Table 1 for the gene sequences. Expression levels for each gene were then normalized to glyceraldehyde 3-phosphate dehydrogenase (GAPDH). The fold change for the ADSC group was set as 1-fold and values in chondrogenic and osteogenic groups were normalized with respect to that of the ADSC group.

**Table 1:** Primer information for RT-qPCR.

Gene		Primer
Aggrecan	Forward	5′-TCCCCTGCTATTTCATCGAC-3'
	Reverse	5′-CCAGCAGCACTACCTCCTTC-3'
SOX9	Forward	5′-AGCGAACGCACATCAAGAC-3'
	Reverse	5′-CTGTAGGCGATCTGTTGGGG-3'
COL2A1	Forward	5′-CCAGATGACCTTCCTACGCC-3'
	Reverse	5′-TTCAGGGCAGTGTACGTGAAC-3'
COL1	Forward	5′-CAGAACGGCCTCAGGTACCA-3'
	Reverse	5′-CAGATCACGTCATCGCACAAC-3'
BSP	Forward	5′-AACGAAGAAAGCGAAGCAGAA-3'
	Reverse	5′-TCTGCCTCTGTGCTGTTGGT-3'
RUNX2	Forward	5′-GGTTAATCTCCGCAGGTCACT-3'
	Reverse	5′-CACTGTGCTGAAGAGGCTGTT-3'
ALP	Forward	5′-AGCTGAACAGGAACAACGTGA-3’
	Reverse	5′-CTTCATGGTGCCCGTGGTC-3’
GAPDH	Forward	5′-CACATGGCCTCCAAGGAGTA-3’
	Reverse	5′-GTACATGACAAGGTGCGGCT-3’

### Bioprinting of the OC interface

Prior to bioprinting, sodium alginate, sodium citrate and CaCl_2_ (Sigma Aldrich, MO) were sterilized with ultraviolet (UV) light for 30 min. Solutions, including 1% (w/v) sodium alginate, 4% (w/v) CaCl_2_ and 4% (w/v) sodium citrate, were prepared by dissolving the powder in sterile deionized (DI) water and subjected to magnetic stirring until the powder was completely dissolved^26^. In order to 3D bioprint the OC interface, the AAB technique was used as described in our recent study^26^. Briefly, alginate droplets were deposited onto 35 × 10 mm tissue culture dishes (Corning, NY) using a micro-valve (INKX0517500, Lee Company, TN) equipped with 250 µm nozzles (INZA3100914K, Lee Company, CT). A unipolar wave pulse with a dwell voltage (amplitude) of 5 V and a dwell time (valve opening duration) of 1000 µs was used for actuating the micro-valve dispenser, where a positive back pressure of ~ 103 kPa was used for driving the flow of sodium alginate inside the tubing. CaCl_2_ vapor was fumed onto alginate for partial crosslinking purposes. In order to fabricate custom-made glass pipettes, borosilicate Pasteur pipettes (VWR, PA) were pulled using P-2000 Flaming/Brown micropipette puller (Sutter Instrument, Novato, CA). Using ~ 80 µm glass pipettes, spheroids were then lifted from the reservoir via aspiration at their critical back-pressure (74 and 95 mmHg for chondrogenic and osteogenic spheroids, respectively), which was controlled by 1/4” NPT 12VDC normally-closed solenoid valves (USS2-00051, U.S. Solid, OH). They were then transferred and positioned onto the alginate support. The procedure was repeated as many times as needed in order to build the construct. At the last step, bioprinted constructs were overlaid with alginate using micro-valve bioprinting and CaCl_2_ vapor was then applied to crosslink alginate. After 1-week culture in hybrid media (chondrogenic and osteogenic media mixed in 1:1 ratio) spheroids were completely fused and alginate was de-crosslinked using 4% sodium citrate solution, leaving the fused OC interface behind.

### Histomorphological analysis of the bioprinted OC interface

The bioprinted OC interface samples were then fixed, embedded in paraffin, and then sectioned for histological analysis. Samples were sectioned transversely in chondrogenic and osteogenic layers separately. H&E Staining was performed according to the protocols explained in Histological analysis section. Sagittal sectioning was also performed across the osteochondral interface and sections were stained for Toluidine Blue O and Alizarin Red S using the protocols described in Immunofluorescence study section. For quantification of cartilage and bone zones, total cartilage or bone area was first measured by selecting the entire region of interest using the area tool in OsteoMeasure software (OsteoMetrics Inc., GA, USA), while the stained areas were selected with the thresholding tool. The thresholding selection was set by sampling from the most darkly stained region of either bone or cartilage, then smoothing/filtering the selection. Any selected areas that were not in the region of interest were deselected before gathering the total stained area. The percentage of stained areas in the bioprinted OC interface were calculated as below:

1 $$\% {\text{of}}\,{\text{stained}}\,{\text{area}} = \frac{{{\text{Stained}}\,{\text{area}}}}{{{\text{Total}}\,{\text{area}}\,{\text{of}}\,{\text{bone}}\,{\text{or}}\,{\text{cartilage}}\,{\text{zone}}}} \times 100$$

### Statistical analysis

All data were presented as the mean ± standard deviation unless stated otherwise, and were analyzed using Student’s t-test and one-way analysis of variance (ANOVA) to test for significance when comparing the data^40^. Post-hoc Tukey’s multiple comparison test was used to determine the individual differences among the groups. Differences were considered significant at *p* < 0.05 (*), *p* < 0.01 (**), and *p* < 0.001 (***). All statistical analysis was performed by Statistical Product and Service Solutions software (SPSS, IBM, USA).
